# FAM64A contributes to ovarian cancer proliferation and metastasis by suppressing TWIST1 ubiquitination and degradation

**DOI:** 10.1530/ERC-24-0048

**Published:** 2025-06-16

**Authors:** Juan Zhao, Ting Yang, Sijuan Tian, Meili Pei, Minyi Zhao, Li Wang, Xiaofeng Yang

**Affiliations:** Department of Gynaecology and Obstetrics, The First Affiliated Hospital of Xi’an Jiaotong University, Xi’an, Shanxi Province, China

**Keywords:** FAM64A, ovarian cancer, TWIST1, ubiquitination, metastasis

## Abstract

Ovarian cancer is among the most common cancers among gynecological malignancies. FAM64A is associated with various cancer progressions, but its function and mechanism in ovarian cancer remain unclear. We analyzed and examined the expression of FAM64A in ovarian cancer cells and tissues. Proliferation, migration and invasion were assessed by knocking down and overexpressing FAM64A in A2780 and SKOV3 cells, respectively. Bioinformatics combined with molecular experiments validated the molecular mechanism of FAM64A. A xenograft tumor model and lung metastasis model were created to explore the impact of FAM64A on tumor growth and metastasis in nude mice. To evaluate the relative signaling molecule expression, immunohistochemistry (IHC) and western blot assays were conducted. FAM64A was upregulated in ovarian cancer tissues and cells and was demonstrated to promote the proliferation, migration and invasion of A2780 and SKOV3 cells *in vitro*. Bioinformatics and western blot assays indicated that FAM64A could regulate the EMT-related transcription factor TWIST1 by suppressing TWIST1 ubiquitination and degradation via the E3 ubiquitin ligase STUB1. Moreover, the knockdown of FAM64A inhibited tumor growth in xenograft tumor mice and lung metastasis *in vivo*. FAM64A exerts its oncogenic function by regulating TWIST1 ubiquitination and degradation, indicating that FAM64A may provide a promising therapeutic target for the treatment of ovarian cancer.

## Introduction

Ovarian cancer (OC) is among the deadliest gynecological malignancies (Vaughan *et al.* 2011). Its high mortality rate is mainly because the majority of patients already have metastases during tumor recurrence, as residual tumors after surgery can stimulate metastatic and invasive cancer patterns (Nick *et al.* 2015, Sapiezynski *et al.* 2016). Despite advances in detecting and treating OC, ineffective targeted therapies other than chemotherapy result in poor prognosis. Therefore, developing new therapeutic targets and biomarkers for detecting OC and response to treatment is attracting attention.

The FAM64A gene is localized on 17p13.12 of the human chromosome. Its protein contains 238 amino acids and is also known as the PICALM-interacting mitotic regulator (PIMREG), originally thought to be a CALM-interacting protein expressed in mitogen responses (Stelzer *et al.* 2016). The initial studies on FAM64A were related to leukemia (Archangelo *et al.* 2006). The expression of FAM64A affects the cellular localization of CALM/AF10 and opposes the activation of leukemia fusion proteins (Archangelo *et al.* 2006). Zhao *et al.* confirmed that the expression level of FAM64A presented periodic changes in the cell cycle and that FAM64A could be used as a substrate for the anaphase-promoting complex/cyclosome (APC/C) in the late cell cycle (Zhao *et al.* 2008). Meanwhile, it has been proposed that the expression of FAM64A protein depends on the cell cycle and is closely related to cell proliferation (Archangelo *et al.* 2008, Kimes *et al.* 2014). Studies have found that FAM64A is prevalent in fetal cardiomyocytes, and its expansion declines precipitously after birth. *In vitro*, the knockdown of FAM64A suppressed fetal cardiomyocyte proliferation, whereas overexpression had the opposite effect (Hashimoto *et al.* 2022). In addition, FAM64A was implicated in facilitating cell proliferation, migration and epithelial-to-mesenchymal transition in many tumor cell lines. FAM64A has been demonstrated to be upregulated in tumor tissues such as triple-negative breast cancer and is associated with poor tumor prognosis (Zhang *et al.* 2014). These studies show that FAM64A is significantly associated with a variety of cancers. However, the underlying role of FAM64A in the diagnosis and prognosis of OC has not been characterized.

Investigations have stated that epithelial-mesenchymal transition (EMT) is a critical aspect of cytokine/growth factor-induced EMT in the tumor microenvironment during tumor progression or metastasis (Chen *et al.* 2023). The EMT-associated transcription factor TWIST1 has been reported to be transcriptionally enabled by various cytokines (IL-6 or TGF-β), thereby triggering EMT in cancer cells (Guan *et al.* 2023). Multiple E3 ubiquitin ligases have been observed to modulate the ubiquitination of TWIST1 and stabilize its expression levels, such as USP29 and RBX1 (Shao *et al.* 2022, Guan *et al.* 2023). In our study, the E3 ubiquitin ligase STUB1 was identified to participate in regulating TWIST1 by FAM64A. Researchers have revealed that STUB1 is implicated in regulating various mechanisms of cancer development, metastasis or drug resistance and may also function as a tumor suppressor by de-deleting oncoproteins, such as in hepatocellular carcinoma and prostate cancer (Liao *et al.* 2023, Liu *et al.* 2023). However, the exact mechanism through which FAM64A stabilizes TWIST1 expression in OC remains unclear. Our experiments may provide essential help for the development of molecular drug studies to inhibit tumor metastasis.

In this study, we found that FAM64A was upregulated in OC tumor tissues and positively correlated with EMT-related transcription factor TWIST1 expression, indicating that FAM64A has vital significance for the development of OC. FAM64A promoted OC cell proliferation, migration and invasion *in vitro*. Consistently, the knockdown of FAM64A inhibited tumor growth and lung metastasis in mice. Further molecular mechanistic studies revealed that FAM64A decreased TWIST1 ubiquitination and degradation via the E3 ubiquitin ligase STUB1 and activation of the Wnt/β-catenin signaling pathway. Therefore, perhaps FAM64A is a meaningful candidate target for OC therapy strategies.

## Materials and methods

### Bioinformatics analysis

We used the Gene Expression Profiling Interactive Analysis (GEPIA) analysis platform (http://gepia.cancer-pku.cn) to retrieve the expression of FAM64A in OC and the correlation between FAM64A and EMT-related factors. In the UbiBrowser analysis platform (http://ubibrowser.bio-it.cn/ubibrowser/), we identified several E3 ubiquitin ligases that regulate FAM64A.

### Clinical samples

Samples of ovarian tissues and normal ovarian tissues from 60 patients with OC who were surgically resected but had not received preoperative radiotherapy, chemotherapy or hormonal treatment at the First Affiliated Hospital of Xi’an Jiaotong University were collected for this study. Approval for this study was granted by the Ethics Committee of the First Affiliated Hospital of Xi’an Jiaotong University, and written informed consent was obtained from each patient.

### Quantitative real-time polymerase chain reaction (qRT-PCR)

TRIzol (15596026, Invitrogen, USA) reagent was used for the isolation of total RNA according to the manufacturer’s instructions, followed by reverse transcription of RNA to complementary DNA (cDNA) using a PrimeScript RT reagent kit (RR047A, Takara, Japan). The qPCR analysis was conducted on an ABI 7500 instrument (Applied BioSystems, USA) with SYBR® Premix ExTaq kit (RR420A, Takara). The primer sequences were as follows: FAM64A, forward: 5′- CCAGAAAGCTAGGTCGTGGGT-3′ and reverse: 5′- TGGACTGATCGTGCTTCGTGT-3′; TWIST1, forward: 5′-GGCTCAGCTACGCCTTCTC-3′ and reverse: 5′-TCCTTCTCTGGAAACAATGACA-3′); GAPDH, forward: 5′-ACCCAGAAGACTGTGGATGG-3′ and reverse: 5′-TCTAGACGGCAGGTCAGGTC-3′. GAPDH acted as an internal reference. RNA expression levels were calculated using the 2^−ΔΔCt^ method.

### Western blot assay

Total protein from tissues and cells was extracted using radioimmunoprecipitation assay (RIPA) lysis buffer (P0013B, Beyotime, China), and the protein concentration was determined by bicinchoninic acid (BCA) kit (P0009, Beyotime, China). The protein was then separated by 10% sodium dodecyl sulfate-polyacrylamide gel electrophoresis (SDS-PAGE) and electrically transferred onto polyvinylidene difluoride (PVDF) membranes. The membranes were blocked using 5% skim milk for 2 h and then incubated overnight at 4°C with the subsequent primary antibodies against FAM64A (1:200, Cat#NBP3-03210, Novus Biologicals, USA), TWIST1 (1:500, Cat#ab175430, Abcam, USA), E-cadherin (1:1,000, Cat#ab133597, Abcam), N-cadherin (1:5,000, Cat#ab76011, Abcam), ZEB1 (1:1,000, Cat#ab303480, Abcam), ZEB2 (1:2,000, Cat#ab191364, Abcam), Snail1 (1:20,000, Cat#ab229701, Abcam), Slug (1:1,000, Cat#ab180714, Abcam), STUB1 (1:10,000, Cat#ab134064, Abcam), GSK-3β (1:500, Cat#ab93926, Abcam), β-catenin (1:5,000, Cat#ab32572, Abcam), GAPDH (1:500, Cat#ab8245, Abcam) and Lamin B1 (1:500, Cat#ab16048, Abcam). After that, the membranes were incubated with the corresponding HRP-conjugated secondary antibodies (anti-mouse, 1:1,000, Cat#ab6728, Abcam; anti-rabbit, 1:1,000, Cat#ab6721, Abcam) at room temperature for 1 h. Afterward, the protein expressions were visualized and observed by an enhanced chemiluminescence reagent (ECL) kit (P0018S, Beyotime, China). The intensity of protein bands was analyzed using ImageJ software. GAPDH and Lamin B1 were used as internal controls. Proteins that are situated in the cytoplasm or nucleus were separated using the Cytoplasmic and Nuclear RNA Purification Kit (Norgen, Canada) in accordance with the manufacturer’s instructions.

### Cell culture and transfection

We used the endometrioid OC line A2780, the likely ovarian clear cell carcinoma (OCCC) line SKOV3, the OCCC line TOV-21G, the high-grade serous OC cell lines CAOV3 and OVCAR4, and the human normal ovarian epithelial cell line (IOSE80) (Anglesio *et al.* 2013, Domcke *et al.* 2013). Cell lines were sourced from the American Type Culture Collection (ATCC) and cultivated in a 5% CO_2_ environment filled with 10% (v/v) FBS (Gibco, USA), 100 U/mL penicillin and 100 μg/mL streptomycin in Dulbecco’s modified Eagle’s medium (DMEM, Invitrogen, USA). The FAM64A sequences (forward: 5′-GGCUCAUGCCCACCCAUTT-3′, reverse: 5′-AUGGGUGGGCAUGUGAGCCTT-3′) were inserted into the pc-DNA3.1 vector to overexpress FAM64A (FAM64A), and the control pc-DNA3.1 empty vector (Vector), a lentiviral vector containing short hairpin RNA against FAM64A (sh-FAM64A) and negative control (sh-NC) were all constructed by Guangzhou RiboBio Co., Ltd (China). Next, Lipofectamine 3000 (Invitrogen) was used to perform cell transfection based on the manufacturer’s instructions.

### Cell counting kit-8 (CCK-8) assay

Cells (2 × 10^3^ cells/well) were inoculated into a 96-well plate and plated for indicated days. Next, 10 μL of CCK8 solution (C0042, Beyotime, China) was added and incubated for 4 h. The optical density value recorded at 450 nm was determined with a microplate reader.

### Colony formation assay

The transfected cells (600 cells/well) were seeded into a 6-well plate and incubated continuously for 2 weeks. The colonies were fixed with 4% paraformaldehyde, washed with PBS, stained with 0.5% crystal violet, photographed and counted.

### Transwell assay

Cell migration and invasion experiments were referred to in the previous study (Zhang *et al.* 2019*b*). In brief, the upper chamber of transfected cells (1 × 10^4^ cells/well) was inoculated in 100 μL of serum-free medium and the lower chamber was filled with 600 μL of complete medium containing 10% FBS. After 24 h, cells transferring to the surface of the lower chamber were fixed with 4% paraformaldehyde and stained with 0.5% crystal violet. The stained cells were imaged and counted under an inverted microscope (Olympus, Japan). Multiple fields of view were randomly selected under the microscope, and the number of cells in each field was counted and averaged.

### Co-immunoprecipitation (Co-IP)

Co-IP analysis, as mentioned in the previous studies (Shao *et al.* 2022), in short, A2780 cell lysates (500 μg) were prepared in ice-cold RIPA buffer, centrifuged and incubated overnight at 4°C with anti-FAM64A antibody (2 μg) and protein G agarose beads (20 μL). IgG was used as a negative control. Bound proteins were eluted with RIPA buffer, followed by boiling in SDS sample buffer and expression of TWIST1 and STUB1 was detected by western blot.

### Ubiquitination assays

Ubiquitination assays, as mentioned by Xie *et al.* (2022), in brief, A2780 cells transfused with HA-Ub were processed with MG132 (20 μM) for 8 h. After incubation of the lysates with the anti-TWIST1 antibody for 3 h, protein A/G agarose beads were incorporated for 8 h at 4°C. Reactions were conducted on immunoblotting (IB) analysis.

### Immunohistochemistry (IHC) assay

After formalin fixation and paraffin embedding, we prepared 4 μm-thick sections of the collected tissues. The sections were processed according to the study of Zhang & Guo (2022). Subsequently, the sections were then incubated with primary antibodies against FAM64A (1:50, Cat#NBP3-03210, Novus Biologicals, USA), TWIST1 (1:200, Cat#ab175430, Abcam) and Ki-67 (1:500, Cat#ab192742, Abcam) at 4°C overnight, followed by incubation with peroxidase-conjugated antibody for 30 min at 37°C. After that, the sections were visualized using 3,3-diaminobenzidine (PR30010, DAB, Proteintech, China) for 10 min. Then, the sections were counterstained with hematoxylin, dehydrated and the cover slipped after washing. The images were obtained and photographed using a light microscope (Olympus, Japan). IHC scores are categorized into the following four levels: 0 (negative), 1 (weakly positive), 2 (moderately positive) and 3 (strongly positive).

### *In vivo* experiments

Female BALB/c nude mice (4–6 weeks old) were obtained from Beijing Weitong Lihua Experimental Animal Technology Co., Ltd For xenograft tumor models, nude mice were classified randomly into two groups (*n* = 6 per group). 1 × 10^6^ SKOV3 cells stabilized with sh-FAM64A or sh-NC suspended in 200 μL normal saline were subcutaneously injected into the right flank of mice. The tumor volume was measured every 5 days with a vernier caliper. The tumor volume was calculated using the formula: length × width^2^ × 0.5. After 25 days of injection, the mice were sacrificed and the tumor tissues were excised for weighing and expression analysis. For lung metastasis models, nude mice were randomly divided into two groups (*n* = 3 per group). 1 × 10^6^ SKOV3 cells transfected with sh-FAM64A or sh-NC were injected into nude mice through the caudal vein. After 5 weeks, the mice were sacrificed and the lung tissues were stained with hematoxylin-eosin. The Animal Care and Use Committee of the First Affiliated Hospital of Xi’an Jiaotong University approved all animal experiments.

### Statistical analysis

The experimental data were represented as means ± standard deviation. GraphPad 7 software package (USA) was used for graphing and statistical analyses. The statistical comparisons between two groups were analyzed using Student’s *t*-test and the comparisons of more than two groups were evaluated using one-way analysis of variance (ANOVA). Pearson chi-square test was performed to analyze the correlation between the expression of FAM64A and clinicopathological characteristics. Spearman’s correlation analysis was applied to evaluate the expression correlations between FAM64A and TWIST1. The difference was considered statistically significant when *P* < 0.05.

## Results

### FAM64A is highly expressed in OC tissues and cells

**Figure 1:**
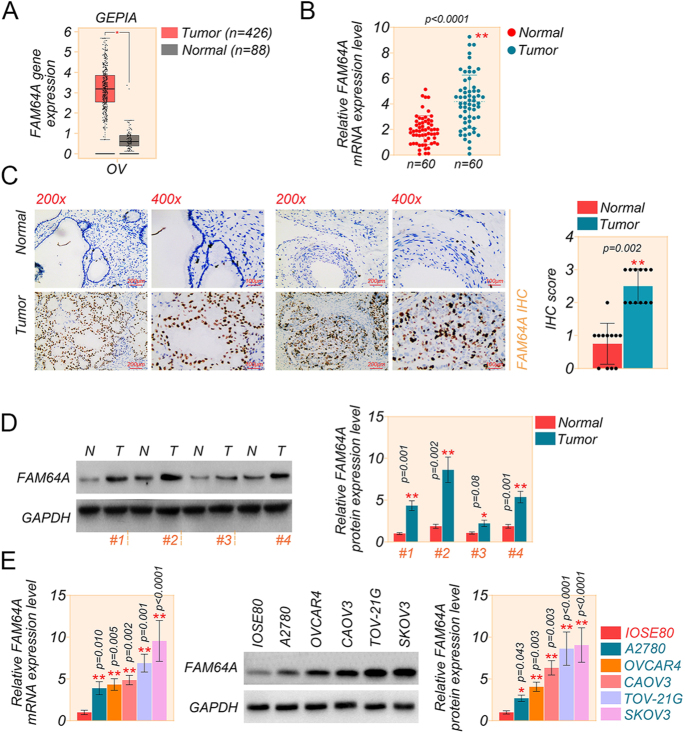
FAM64A is up-regulated in OC tissues and cells. (A) GEPIA (http://gepia.cancer-pku.cn) analysis of FAM64A expression in TCGA cohort high-throughput RNA sequencing data of OC. (B) The mRNA expression of FAM64A in 60 OC tissues and normal tissues was determined by qRT-PCR assay. (C) IHC staining detected the expression of FAM64A in five OC tissues and normal tissues (scale bar = 200/100 μm, magnification, 200×/400×). (D) Representative image of the expression level of FAM64A protein in four OC and normal tissues by western blot assay. (E) The mRNA and protein level of FAM64A in human OC cell lines (A2780, OVCAR4, CAOV3, TOV-21G, SKOV3) and human normal ovarian epithelial cell line (IOSE80) was determined by qRT-PCR and western blot assays. *T*-test for two comparisons and one-way ANOVA for comparisons of three or more groups. Data are shown as mean ± SD. *P < 0.05, **P < 0.01. A full color version of this figure is available at https://doi.org/10.1530/ERC-24-0048.

Previous analysis on the Gene Expression Omnibus (GEO) platform (GSE18520) revealed high expression of FAM64A in OC, yet overall survival (OS) was positively correlated with expression (Zheng *et al.* 2023). Therefore, we revalidated the high level of FAM64A expression in OC tissues by the GEPIA dataset of high-throughput RNA sequencing data from The Cancer Genome Atlas (TCGA) cohort (Fig. 1A). FAM64A was more abundant in high-grade serous OC and endometrioid carcinoma tissues of 60 OC patients than in normal ovarian tissues by qRT-PCR assays (Fig. 1B and Supplementary Fig. S1A (see section on Supplementary materials given at the end of the article)). In addition, FAM64A expression was determined in OC samples and normal ovarian tissues (which did not receive preoperative radiotherapy or chemotherapy) by IHC staining (Fig. 1C) and western assays (Fig. 1D). The data showed that FAM64A was higher in high-grade serous OC and endometrioid carcinoma tissues and lower in normal ovarian tissues (Supplementary Fig. S1B). We presented the basic information of these 60 patients in Supplementary Table S1. Meanwhile, the upregulated expression of FAM64A was also found in human OC cell lines (A2780, OVCAR4, CAOV3, TOV-21G, SKOV3) compared with human normal ovarian epithelial cell line (IOSE80), as measured by qRT-PCR and western assays (Fig. 1E). These findings prompted that FAM64 is highly expressed in OC.

### FAM64A facilitates the expansion of OC cells

**Figure 2:**
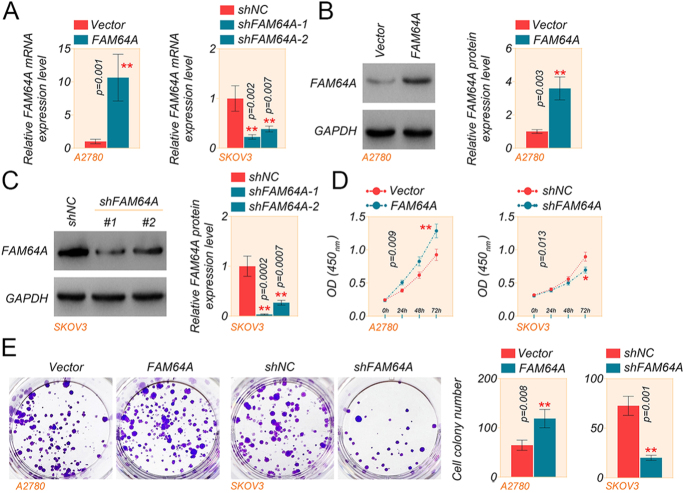
FAM64A promotes the proliferation of OC cells. (A) The mRNA expression of FAM64A in A2780 and SKOV3 cells was measured by qRT-PCR assay. (B and C) The protein level of FAM64A in A2780 and SKOV3 cells was determined by western blot assay. (D) CCK-8 assay was used to determine the cell viability of A2780 and SKOV3 cells. (E) A colony formation assay detected the number of cell clones in A2780 and SKOV3 cells. Statistical analyses of the CCK-8 experiment results were analyzed using a two-way ANOVA, *t*-test for two comparisons and one-way ANOVA for comparisons of three or more groups. Data are shown as mean ± SD. *P < 0.05 and **P < 0.01. A full color version of this figure is available at https://doi.org/10.1530/ERC-24-0048.

To characterize the biofunction of FAM64A in OC cells, we measured the efficiency of knockdown or overexpression of FAM64A in A2780 and SKOV3 cells, as illustrated in Fig. 2A, B, C. Since sh-FAM64A-1 was more efficient in gene silencing in SKOV3 cells, we picked sh-FAM64A-1 (labeled as sh-FAM64A) for further functional experiments. CCK-8 and colony formation assays revealed that FAM64A overexpression dramatically increased the proliferation of A2780 cells, whereas FAM64A reduction markedly hindered the proliferation of SKOV3 cells (Fig. 2D and E).

The A2780 and SKOV3 cell lines have long been used as the most utilized cell lines in OC research. Among them, A2780 cells are used as ovarian endometrioid adenocarcinomas, while SKOV3 cells were previously considered high-grade ovarian serous adenocarcinoma. Some studies have pointed out that the characteristics of SKOV3 cells are more consistent with those of ovarian clear cell adenocarcinoma (Domcke *et al.* 2013). Therefore, we again verified the role of FAM64A in the OVCAR4 cell line, which belongs to high-grade ovarian serous adenocarcinoma. Subsequently, we also observed the effect of FAM64A knockdown on cell proliferation in OVCAR4 cells. The results indicated that FAM64A knockdown decreased FAM64A expression in OVCAR4 cells and inhibited cell proliferation (Supplementary Fig. S2A, B, C). Taken together, FAM64A stimulates the proliferation of OC cells *in vitro*.

### FAM64A encourages the migration and invasion of OC cells

**Figure 3:**
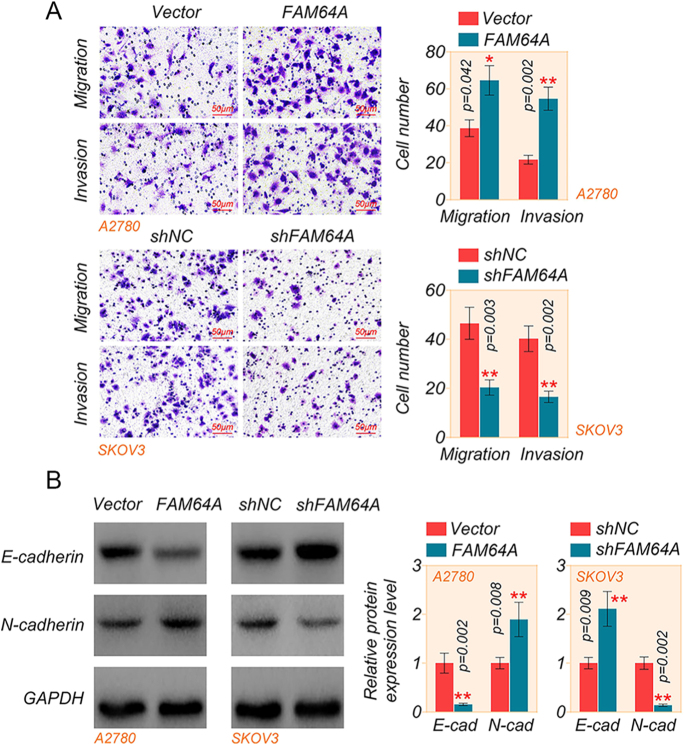
FAM64A promotes the migration and invasion of OC cells. (A) Transwell assay was used to detect the migration and invasion capabilities of A2780 and SKOV3 cells (scale bar = 200 μm, magnification, 100×). (B) The levels of E-cadherin and N-cadherin in A2780 and SKOV3 cells were measured by western blot assay. *T*-test. Data are shown as mean ± SD. *P < 0.05 and **P < 0.01. A full color version of this figure is available at https://doi.org/10.1530/ERC-24-0048.

The migration and invasion transwell assays demonstrated that overexpression of FAM64A increased cell migration and invasion in A2780 cells, while knockdown of FAM64A inhibited cell migration and invasion in SKOV3 cells (Fig. 3A). Western blot analysis visualized that FAM64A overexpression diminished E-cadherin expression and amplified N-cadherin expression in A2780 cells. In contrast, the knockdown of FAM64A exhibited the opposite result in SKOV3 cells (Fig. 3B). Similarly, FAM64A knockdown reduced migratory invasive ability and EMT in OVCAR4 cells (Supplementary Fig. S2D and E). These data suggested that FAM64A accelerates migration, invasion and EMT of OC.

### FAM64A is positively associated with the EMT-related transcription factor TWIST1

**Figure 4:**
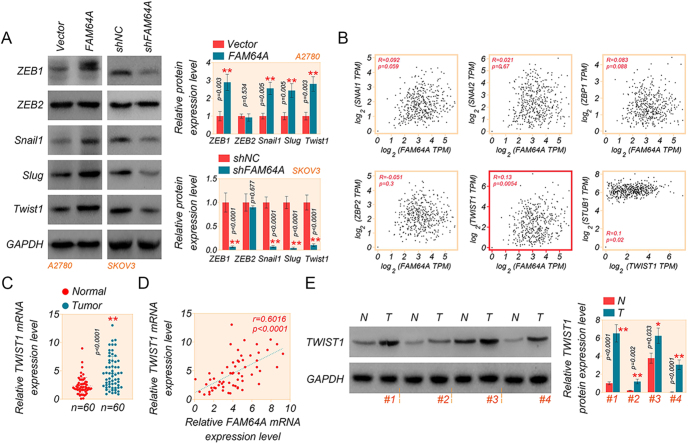
FAM64A is positively associated with the EMT-related transcription factor TWIST1. (A) The levels of EMT-related transcription factors in A2780 and SKOV3 cells were measured by western blot assay. (B) GEPIA analysis of the correlation between FAM64A and EMT-related transcription factors in OC tissues. (C) The mRNA expression of TWIST1 in 60 OC tissues and normal tissues was determined by qRT-PCR assay. (D) The correlation between the expression of FAM64A and TWIST1 was analyzed in the OC tissues of 60 patients by qRT-PCR assay. (E) The expression level of TWIST1 protein in four OC and normal tissues by western blot assay. *T*-test. Data are shown as mean ± SD. *P < 0.05 and **P < 0.01. A full color version of this figure is available at https://doi.org/10.1530/ERC-24-0048.

To investigate the association of FAM64A and EMT, we checked a series of EMT-related transcription factors by western assays (Fig. 4A). The results visualized that FAM64A overexpression increased the expression of ZEB1, Snail1, Slug and TWIST1, and FAM64A knockdown showed an opposite effect in SKOV3 cells. In particular, the most significant change was in TWIST1 expression; the expression level of ZEB2 did not change significantly in any of the above treatments (Fig. 4A).

Besides, the GEPIA database analysis also found a positive correlation between FAM64A and EMT-related factor TWIST1 (*r* = 0.13, *P* = 0.0054) in OC tissues (Fig. 4B). However, there was no correlation between FAM64A and several other genes, although the correlation was weak (Fig. 4B). To further confirm the significance of ZEB1, Snail1, Slug, and TWIST1 in OC, we examined their expression levels using qRT-PCR assays. TWIST1 was highly expressed in high-grade serous tissues but not statistically significant in endometrioid carcinoma of OC patients compared with normal ovarian tissues (Supplementary Fig. S1C). We discovered that a linear correlation positively associated with the expression of FAM64A and TWIST1 (*r* = 0.5835, *P* < 0.0001) was also confirmed in the high-grade serous OC patients (Fig. 4C and D). However, there was no correlation between FAM64A and TWIST1 in ovarian endometrioid carcinoma (Supplementary Fig. S1D). There was no correlation with ZEB1, ZEB2 and Snail1 (Fig. 4C and D, and Supplementary S1D). FAM64A correlates with neither ZEB1, ZEB2 nor Snail1 in high-grade serous OC and endometrioid carcinoma (Supplementary Fig. S1D). Consistently, the expression of TWIST1 was higher in OC samples than in normal ovarian tissues using western assays (Fig. 4E).

### FAM64A promotes TWIST1 expression in OC cells via STUB1

**Figure 5:**
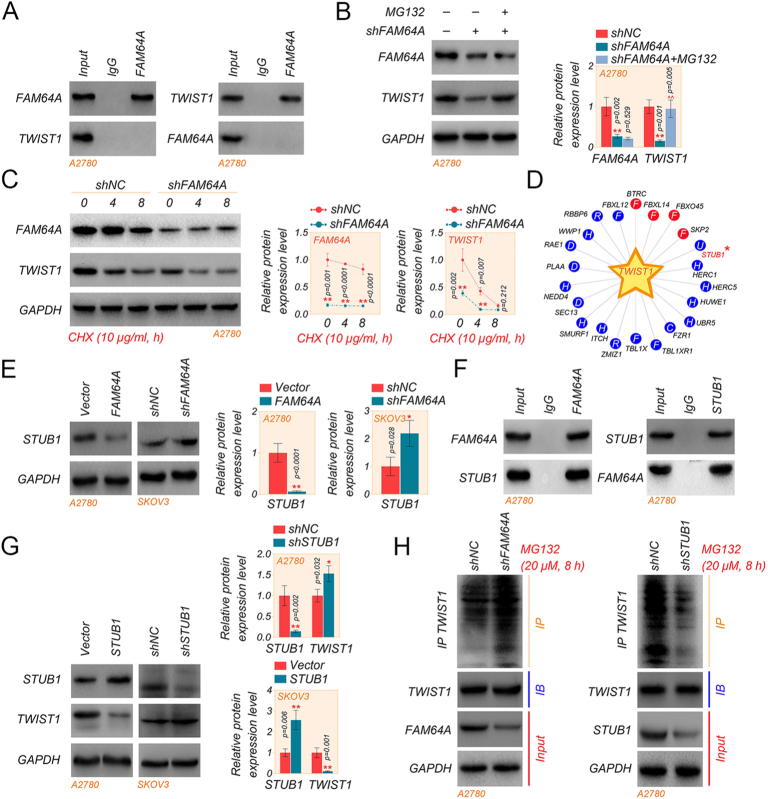
FAM64A promotes TWIST1 expression in OC cells via STUB1. (A) Co-IP assay detected the interaction between FAM64A and TWIST1. (B and C) The expression level of FAM64A and TWIST1 was measured in FAM64A-deficient A2780 cells treated with MG132 (20 μM, 8 h) or CHX (10 μg/mL, h). (D) UbiBrowser v3 was used to predict the E3 ubiquitin ligase that regulates TWIST1. (E) The protein level of STUB1 in A2780 and SKOV3 cells was determined by western blot assay. (F) Co-IP assay detected the interaction between FAM64A and STUB1. (G) The protein level of STUB1 and TWIST1 in A2780 and SKOV3 cells was determined by western blot assay. (H) TWIST1 ubiquitination in the HEK293 cells was detected. The indicated A2780 cells were co-transfected with shFAM64A or Flag-STUB1, treated with MG132 (20 μM, 8 h), and cell lysates were immunoprecipitated with anti-TWIST1 antibody by western blot. *T*-test for two comparisons and one-way ANOVA for comparisons of three or more groups. Data are shown as mean ± SD. *P < 0.05 and **P < 0.01. A full color version of this figure is available at https://doi.org/10.1530/ERC-24-0048.

To further clarify the relationship between FAM64A and TWIST1, we examined whether FAM64A and TWIST1 interacted with each other using a Co-IP assay. The outcomes indicated that there was an absence of direct interaction between FAM64A and TWIST1 (Fig. 5A). Considering that FAM64A can inhibit the expression of TWIST1, we repressed the expression of FAM64A and observed the expression of TWIST1 using MG132 or CHX treatment in A2780 cells. It was recognized that TWIST1 expression was downregulated in the shFAM64A group, while the treatment with MG132 resulted in the upregulation of TWIST1 expression (Fig. 5B). The expression level of TWIST1 was gradually downregulated by CHX treatment and decreased more significantly in the shFAM64A group (Fig. 5C).

Therefore, we suspected that FAM64A might stabilize TWIST1 expression by regulating ubiquitination. Then, UbiBrowser v3 (http://ubibrowser.bio-it.cn/) was used to predict the E3 ubiquitin ligase that regulates TWIST1, and STUB1 (STIP1 homology and U-box containing protein 1) had the highest confidence score (Fig. 5D). STUB1 has been reported to contribute to protein ubiquitination and degradation in cancers (Zhao *et al.* 2022), and we found that FAM64A inhibited STUB1 expression (Fig. 5E); there was a direct interaction between FAM64A and STUB1 in our study (Fig. 5F). Finally, we examined STUB1 and TWIST1 in A2780 and SKOV3 cells overexpressing and knocking down STUB1, and STUB1 and TWIST1 have opposite expression levels (Fig. 5G). Meanwhile, either overexpression of STUB1 or knockdown of FAM64A promotes TWIST1 ubiquitination (Fig. 5H). Our results suggested that FAM64A promoted TWIST1 expression likely by repressing TWIST1 ubiquitination modifications through STUB1.

### FAM64A activates the Wnt/β-catenin signaling pathway in OC cells

**Figure 6:**
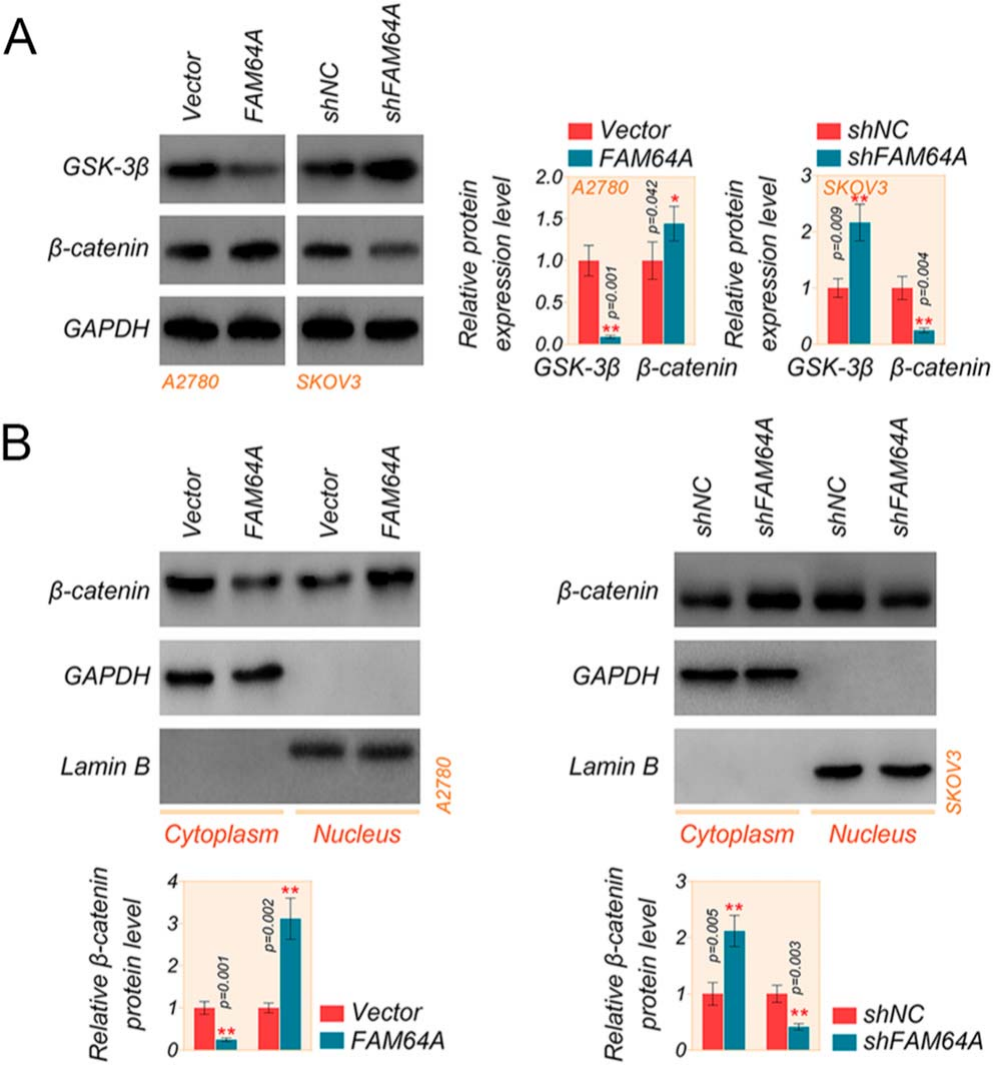
FAM64A activates the Wnt/β-catenin signaling pathway in OC cells. (A) The levels of GSK-3β and β-catenin in A2780 and SKOV3 cells were evaluated by western blot assay. (B and C) The levels of β-catenin in the nucleus and cytoplasm of A2780 and SKOV3 cells were analyzed by western blot assay. *T*-test. Data are shown as mean ± SD. *P < 0.05 and **P < 0.01. A full color version of this figure is available at https://doi.org/10.1530/ERC-24-0048.

Wnt/β-catenin signaling is a driver of epithelial-mesenchymal transformation (EMT), and GSK-3β induces phosphorylation and degradation of β-catenin, which is the main mechanism for regulating β-catenin levels (Arend *et al.* 2013, Zhan *et al.* 2017, Basu *et al.* 2018). The molecular mechanism of the FAM64A/TWIST1 axis in EMT progression was investigated by western blot. We examined the activation of the Wnt/β-catenin signaling pathway in FAM64A overexpression and knockdown, and Supplementary Fig. S3 shows the results of FAM64A transfection validation in A2780 and SKOV3 cells. The findings revealed that overexpression of FAM64A resulted in a decreased GSK-3β level and increased levels of β-catenin as inducers of EMT, while knockdown of FAM64A led to the opposite results (Fig. 6A). A typical marker in the Wnt signaling pathway, β-catenin is a chemical switch that concentrates in the cytoplasm and triggers the transmission of genes targeted for Wnt signaling (Mosimann *et al.* 2009). Our data showed that FAM64A forwardly modulates the expression of β-catenin protein expression, thus we hypothesized that FAM64A could facilitate the accumulation of β-catenin in the cytoplasm and then move it to the nucleus, resulting in the activation of the Wnt/β-catenin signaling pathway. Therefore, we dissociated nuclear and cytoplasmic proteins and realized that overexpression of FAM64A produced a decline in the expression of β-catenin in the cytoplasm and a significant increase in expression in the nucleus. In contrast, the knockdown of FAM64A caused the contrary outcome (Fig. 6B). These data suggested that FAM64A enhances the β-catenin translocation from the cytoplasm to the nucleus and initiates downstream gene transcription. It is conceivable that FAM64A facilitates the progression of OC might be associated with the modulation of TWIST1 and Wnt/β-catenin signaling pathways.

### FAM64A suppression depresses tumor growth and metastasis of OC cells

**Figure 7:**
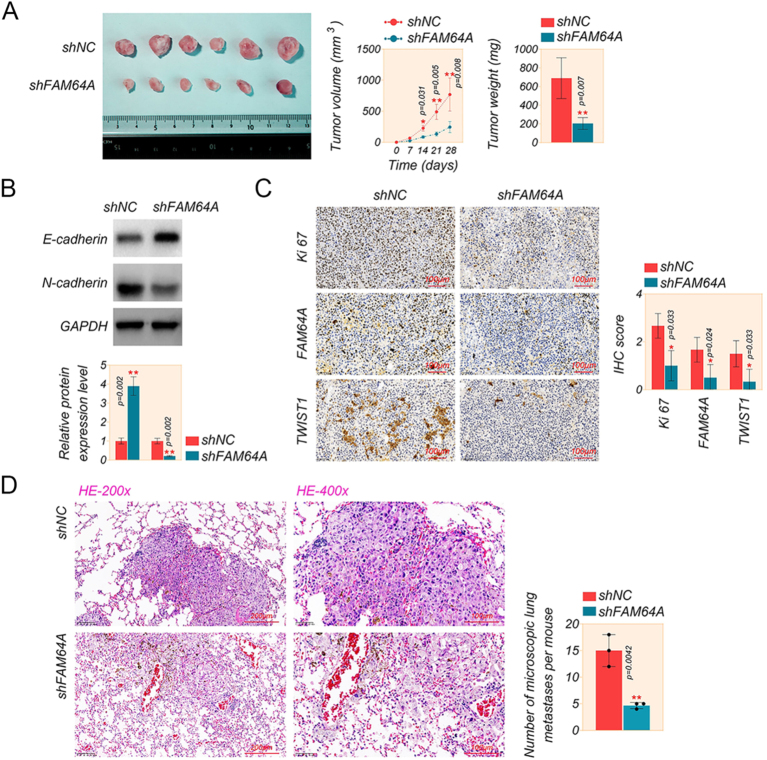
Knockdown of FAM64A inhibits tumor growth and metastasis of OC cells. (A) Photographs of tumors, tumor volume and tumor weight excised from the mice injected with SKOV3 cells transfected with sh-NC or sh-FAM64A. (B) The levels of E-cadherin and N-cadherin in A2780 and SKOV3 cells were measured by western blot assay. (C) The Ki-67, FAM64A and TWIST1 tumors from mice injected with SKOV3 cells were evaluated by IHC staining (scale bar = 100 μm, magnification, 200×). (D) H&E-stained sections of metastatic lung foci (scale bar = 200/100 μm, magnification, 200×/400×). *T*-test. Data are shown as mean ± SD. *P < 0.05 and **P < 0.01. A full color version of this figure is available at https://doi.org/10.1530/ERC-24-0048.

To further estimate the efficacy of FAM64A on the oncogenicity of SKOV3 cells, we constructed an *in vivo* xenograft mouse model. SKOV3 cells stabilized with sh-FAM64A or sh-NC were dermally injected into Balb/c nude mice. The representative image showed evident inhibition of tumor size by sh-FAM64A (Fig. 7A). The growth curve of the tumor revealed that FAM64A reduction significantly slowed OC tumor growth (Fig. 7A). Similarly, there was a significant reduction in tumor weight in the sh-FAM64A group compared to the sh-NC group (Fig. 7A). Consistent with *in vitro* experiments, the downregulation of FAM64A increased the expression of E-cadherin and decreased the expression of N-cadherin (Fig. 7B). Furthermore, IHC staining exhibited that the knockdown of FAM64A reduced the expression of Ki-67, FAM64A and TWIST1 in xenograft tumors (Fig. 7C). Those results illustrated that inhibition of FAM64A suppresses OC growth *in vivo*.

To evaluate the role of FAM64A in tumor metastasis *in vivo*, a lung metastasis model was established by injection of sh-FAM64A transfected SKOV3 cells into the tail vein of nude mice. Hematoxylin and eosin (H&E) staining of lung tissues confirmed that knockdown of FAM64A significantly reduced metastatic lung nodules (Fig. 7D). These data suggested that FAM64A promotes the process of proliferation and metastasis in OC.

## Discussion

In recent years, accumulated studies have shown that FAM64A has been investigated as a prospective biomarker for various cancers, with up-regulated expression levels in breast cancer (Yao *et al.* 2019, Zhang *et al.* 2019*a*), pancreatic cancer (Jiao *et al.* 2019), lung cancer (Mizuno *et al.* 2020), prostate cancer (Zhou *et al.* 2021), osteosarcoma (Jiang *et al.* 2020) and colitis-associated cancer (Xu *et al.* 2019). Notably, Jiao *et al.* found OS and recurrence-free survival (RFS) times in pancreatic cancer patients with high FAM64A expression. Increased expression of FAM64A was an isolated rhythmic risk factor for OS and RFS (Jiao *et al.* 2019). It has been reported that high FAM64A expression is strongly implicated in clinical stage, metastasis and poor prognosis of breast cancer (Zhang *et al.* 2019*a*).

FAM64A was also confirmed to be abnormally expressed in OC tissues, suggesting that FAM64A may play a hand in the advancement of OC (Li *et al.* 2020). Further, by analyzing the GEPIA database, we noticed that FAM64A was significantly increased in OC tissues compared with normal tissues, especially high-grade serous OC. There was a weak positive correlation between the expression of FAM64A and TWIST1. We found a correlation between FAM64A and TWIST1 in high-grade serous OC, yet not in ovarian endometrioid carcinoma, which may be closely related to the small sample size. Subsequently, we also verified the high expression of FAM64A in OC tissues and cells. Clinical data analysis also showed that FAM64A was abundantly expressed in high-grade serous OC and ovarian endometrioid carcinoma. Our results may suggest that FAM64A can be used as a marker for high-grade serous OC and ovarian endometrioid carcinoma, yet validation in other OC subtypes requires a larger sample size. Considering that FAM64A could modulate the expression of E-cadherin and N-cadherin proteins, we analyzed a series of EMT-related regulators using bioinformatics, Co-IP and western blot assays. FAM64A was found to promote the expression of TWIST1, and both were positively correlated in ovarian tissues. We confirmed a positive correlation with the expression of TWIST1 in the OC tissues we collected. And we proposed a new molecular mechanism to regulate the progression of OC.

It has been revealed that knocking down FAM64A depressed cell proliferation and decreased cell migration by impeding epithelial-mesenchymal transformation in breast cancer (Yao *et al.* 2019). FAM64A was also found to enhance the stemness features of breast cancer cells (Zhang *et al.* 2019*a*). FAM64A is an oncogene that promotes osteosarcoma cell proliferation, migration and invasion (Jiang *et al.* 2020). Cui *et al.* determined that knockdown of FAM64A inhibited prostate cancer growth *in vivo* (Zhou *et al.* 2021). In this study, we found for the first time that FAM64A can promote the proliferation, migration and invasion of OC cells *in vitro*. In addition, this study is the first to prove the effects of FAM64A on promoting xenograft tumor growth and lung metastasis.

TWIST1 is an essential helix-loop-helix transcription factor that is one of the master modulators of EMT (Yang *et al.* 2004, Casas *et al.* 2011). TWIST1 is known to be opsonized in several human cancers, as well as OC, and assumes an active role in tumor progression and metastasis through diverse chemical processes (Grither *et al.* 2018). TWIST1 represses E-cadherin expression by conjugating to the E-box of the CDH1 promoter, thereby inducing EMT (Brlek *et al.* 2021). Furthermore, it was revealed that TWIST1 is susceptible to ubiquitination regulation, and its ubiquitination level is essential for regulating the progression of glioblastoma and triple-negative breast cancer (Shao *et al.* 2022, Xie *et al.* 2022). As shown in our study, FAM64A facilitated TWIST1 expression, but not in a directly interactive manner, but was able to inhibit TWIST1 degradation by directly inhibiting E2 ubiquitin ligase STUB1-mediated ubiquitination. In contrast, silencing FAM64A expression enhanced STUB1 expression and contributed to the ubiquitination and degradation of TWIST1. Accordingly, we confirmed that FAM64A suppressed TWIST1 ubiquitination and degradation via the E3 ubiquitin ligase STUB1.

The expression of TWIST1 is also closely related to the Wnt/β-catenin signaling pathway. TWIST1 results in the serine phosphorylation at residue 9 in GSK3β, resulting in ubiquitination and degradation, increasing the stability and nuclear translocation of β-catenin (Li & Zhou 2011). Therefore, FAM64A may promote the activation of the Wnt/β-catenin signaling pathway by increasing the expression of TWIST1. In the present study, we demonstrated that FAM64A decreased GSK-3β levels and increased TWIST1 and β-catenin levels by promoting the accumulation of β-catenin in the cytoplasm, which then transferred to the nucleus, thereby activating the Wnt/β-catenin signaling pathway. These data suggest that the role of FAM64A in promoting OC progression may be through increasing the expression of TWIST1 and activation of the Wnt/β-catenin signaling pathway. However, we still cannot determine whether the regulation of TWIST1 expression by FAM64A through STUB1-mediated TWIST1 ubiquitination is the specific mechanism and whether there are other molecular mechanisms by which FAM64A inhibits OC tumor progression, which still needs further investigation.

## Conclusion

In summary, our present study found that FAM64A promoted proliferation, migration and invasion in high-grade serous OC and ovarian endometrioid carcinoma. Downregulation of FAM64A inhibited tumor growth and lung metastasis in mice, possibly by suppressing TWIST1 ubiquitination and degradation via the E3 ubiquitin ligase STUB1 and activating the Wnt/β-catenin signaling pathway. Our findings indicated that FAM64A could be a promising therapeutic target for OC treatment.

## Supplementary materials





## Declaration of interest

The authors declare that there is no conflict of interest that could be perceived as prejudicing the impartiality of the work reported.

## Funding

This work was supported by the Scitech Program Foundation of Shaanxi Province (Grant/Award Number 2021SF-214) and the Scitech Program Foundation of Shaanxi Province (NO. 2023-JC-YB-683).

## Author contribution statement

Juan Zhao and Xiaofeng Yang designed the experiments. Juan Zhao, Ting Yang, Sijuan Tian, Meili Pei, Minyi Zhao and Li Wang carried them out, analyzed and interpreted the data. Juan Zhao and Xiaofeng Yang prepared the manuscript. All authors have read and approved the manuscript.

## Data availability

The authors declare that all data supporting the findings of this study are available within the paper and any raw data can be obtained from the corresponding author upon request.

## Ethics approval

Ethical approval was obtained from the ethics committee of The First Affiliated Hospital of Xi’an Jiaotong University (Approval no. 2022-316).

## Consent to participate statement

Written informed consent was obtained from a legally authorized representative(s) for anonymized patient information to be published in this article.
