# Supplementary material for: FAM64A contributes to ovarian cancer proliferation and metastasis by suppressing TWIST1 ubiquitination and degradation
Source: Endocr Relat Cancer. 2025 Jun 16;32(6):e240048. doi: 10.1530/ERC-24-0048 (PMC12171025; doi:10.1530/ERC-24-0048)

Figure S1 Clinical data analysis of FAM64A expression in high-grade serous and endometrioid carcinoma

(A) The mRNA expression of FAM64A in high-grade serous, endometrioid carcinoma tissues and normal tissues was determined by qRT-PCR assay. (B) HE and IHC staining of FAM64A in high-grade serous, endometrioid carcinoma tissues and normal tissues (magnification, 200×/400×). (C) The mRNA expression of TWIST1 in high-grade serous and endometrioid carcinoma tissues and normal tissues was determined by qRT-PCR assay. (D) The correlation between the expression of FAM64A and TWIST1, ZEB1, ZEB2, and Snail1 was analyzed in high-grade serous and endometrioid carcinoma tissues of 60 patients by qRT-PCR assay. T-test. Data are shown as mean ± SD. *P < 0.05 and **P < 0.01.

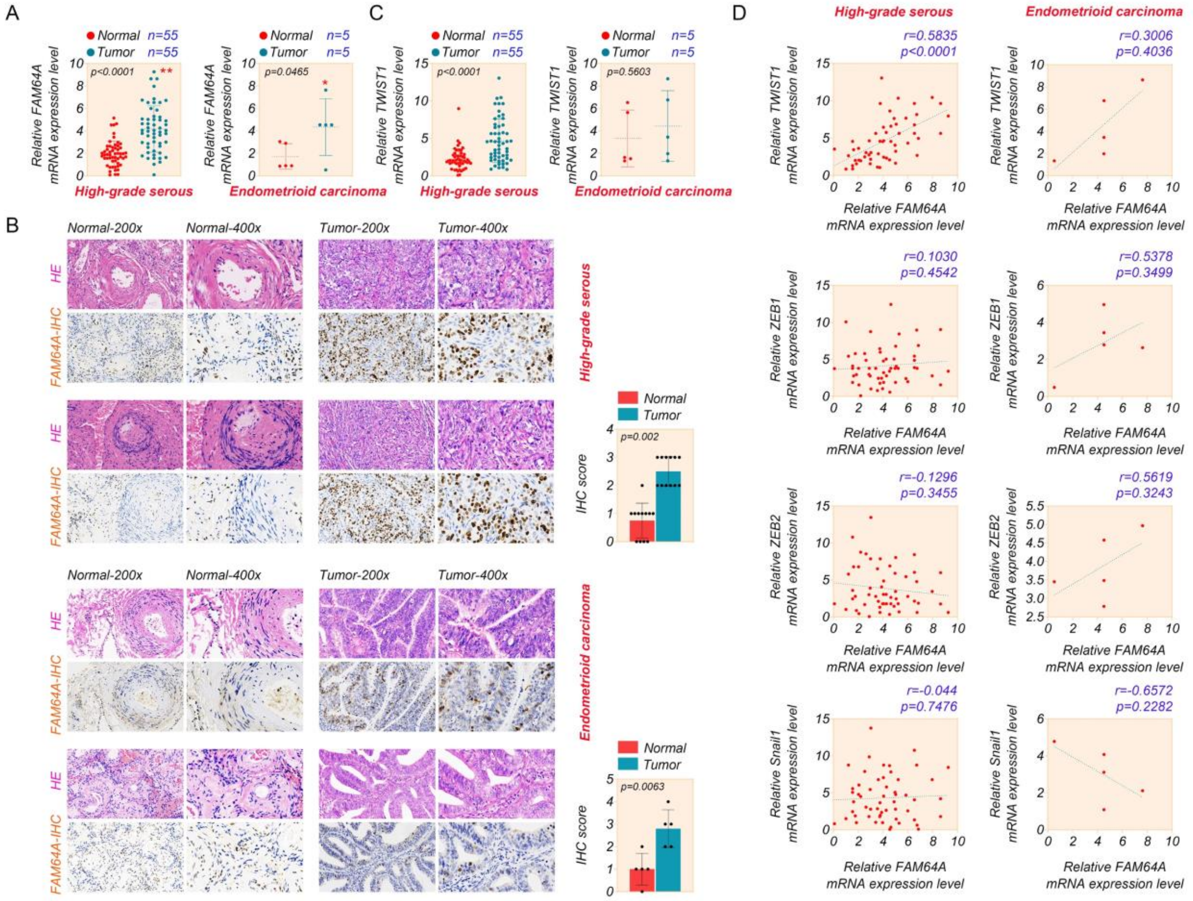

Figure S2 FAM64A promotes the proliferation, migration, and invasion in OVCAR4 cells.

(A)The mRNA and protein expression of FAM64A in OVCAR4 cells were measured by qRT-PCR and WB assays. (B) CCK-8 assay was used to determine the viability of OVCAR4 cells. (C) A colony formation assay detected the number of cell clones in OVCAR4 cells. (D) Transwell assay detected the migration and invasion capabilities of OVCAR4 cells (Scale bar =200 μm, magnification, 100×). (E) The levels of E-cadherin and N-cadherin in OVCAR4 cells were measured by western blot assay. T-test. Data are shown as mean ± SD. *$P < 0.05$ and **$P < 0.01$.

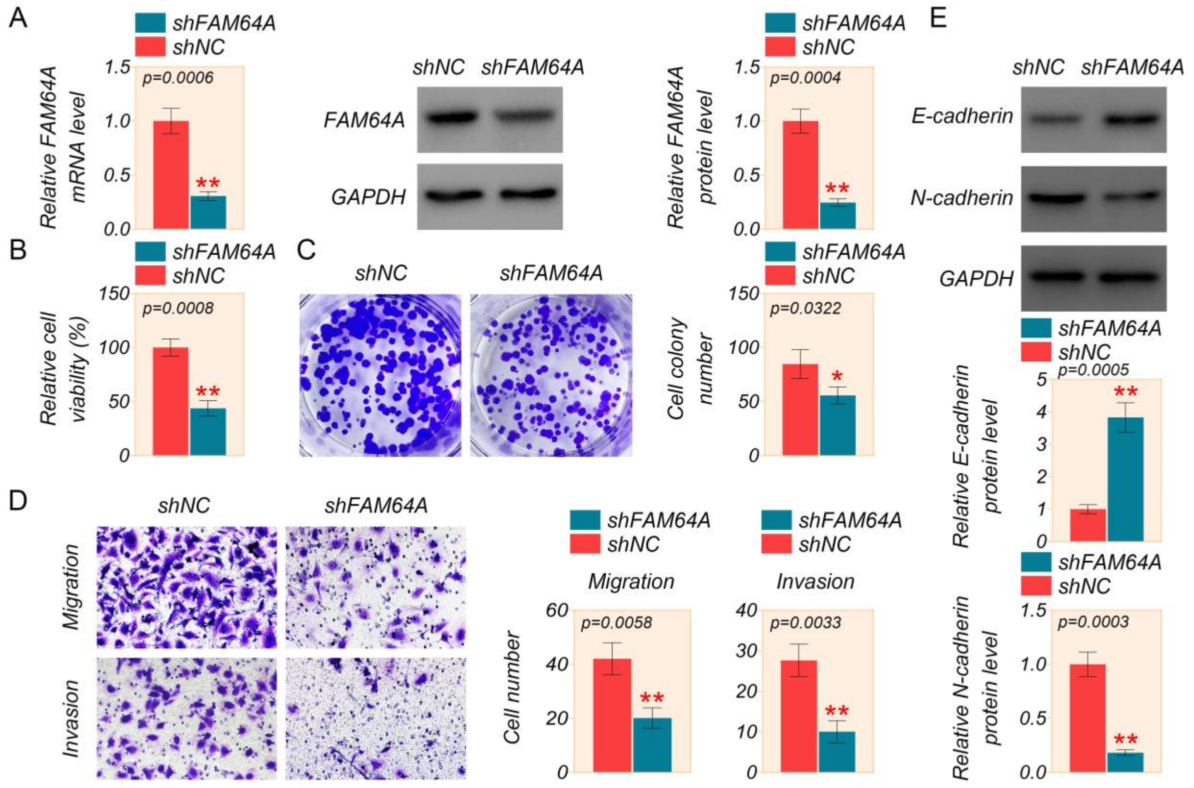

Figure S3 The transfection efficiency of FAM64A in A2780 and SKOV3 cells were measured by qRT-PCR and WB assays. T-test. Data are shown as mean ± SD. $**P < 0.01$.

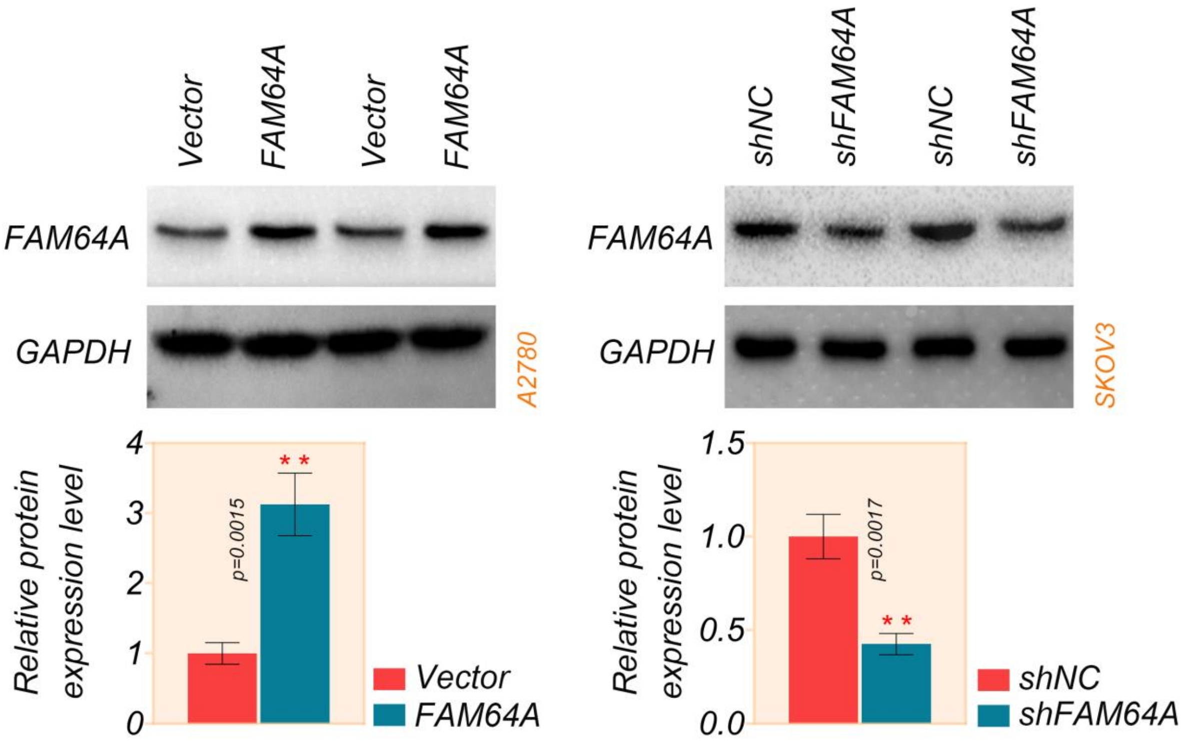

Supplement: Supplementary file 1 [file supplementary_materials.pdf]
